# Sweeteners Show a Plasticizing Effect on PVP K30—A Solution for the Hot-Melt Extrusion of Fixed-Dose Amorphous Curcumin-Hesperetin Solid Dispersions

**DOI:** 10.3390/pharmaceutics16050659

**Published:** 2024-05-15

**Authors:** Kamil Wdowiak, Lidia Tajber, Andrzej Miklaszewski, Judyta Cielecka-Piontek

**Affiliations:** 1 Department of Pharmacognosy and Biomaterials, Poznan University of Medical Sciences, 3 Rokietnicka St., 60-806 Poznan, Poland; kamil.wdowiak@student.ump.edu.pl; 2 School of Pharmacy and Pharmaceutical Sciences, Trinity College Dublin, University of Dublin, D02 PN40 Dublin, Ireland; ltajber@tcd.ie; 3 Institute of Materials Science and Engineering, Poznan University of Technology, Jana Pawla II 24, 61-138 Poznan, Poland; andrzej.miklaszewski@put.poznan.pl

**Keywords:** curcumin, hesperetin, plasticizer, sweeteners, amorphous solid dispersions, hot-melt extrusion, polyvinylpyrrolidone

## Abstract

The co-administration of curcumin and hesperetin might be beneficial in terms of neuroprotective activity; therefore, in this study, we attempted to develop a fixed-dose formulation comprising these two compounds in an amorphous state. The aim of obtaining an amorphous state was to overcome the limitations of the low solubility of the active compounds. First, we assessed the possibility of using popular sweeteners (erythritol, xylitol, and sorbitol) as plasticizers to reduce the glass transition temperature of PVP K30 to prepare the polymer–excipient blends, which allowed the preparation of amorphous solid dispersions via hot-melt extrusion at a temperature below the original glass transition of PVP K30. Erythritol proved to be the superior plasticizer. Then, we focused on the development of fixed-dose amorphous solid dispersions of curcumin and hesperetin. Powder X-ray diffraction and thermal analysis confirmed the amorphous character of dispersions, whereas infrared spectroscopy helped to assess the presence of intermolecular interactions. The amorphous state of the produced dispersions was maintained for 6 months, as shown in a stability study. Pharmaceutical parameters such as dissolution rate, solubility, and in vitro permeability through artificial membranes were evaluated. The best improvement in these features was noted for the dispersion, which contained 15% of the total content of the active compounds with erythritol used as the plasticizer.

## 1. Introduction

Therapies based on compounds of natural origin are an interesting alternative to treatments based on drugs obtained by chemical synthesis. Natural substances can be used in stand-alone therapy or to supplement synthetic medication therapy. One of the well-studied compounds of plant origin is curcumin. This substance is primarily responsible for the known health properties of turmeric (*Curcuma longa* L.) and is found naturally in the rhizome. Curcumin has been shown to have anti-inflammatory, antioxidant, anti-cancer, anti-diabetic, and cardio- and neuroprotective effects [1,2,3,4]. However, its multidirectional effects are limited considerably by its poor water solubility. In recent years, several attempts have been made to improve the biopharmaceutical properties and, thus, the bioavailability of curcumin. Some recent studies on improving the solubility of curcumin involved co-crystal formation with ascorbic acid [5], fabrication of pectin-tannic acid-coated core–shell nanoparticles with curcumin [6], cyclodextrin-based nanosponges of curcumin [7], and fast-dissolving curcumin/cyclodextrin electrospun nanofibrous webs [8].

Another natural compound with potential pharmaceutical uses is hesperetin. The greatest amount of this flavonoid-like substance is found in citrus fruits [9]. Anti-inflammatory, antioxidant, anti-cancer, anti-diabetic, and neuroprotective activities have been proven for hesperetin [10,11,12,13]. Its effective use as a pro-healthy agent is hampered by poor solubility. There have been many attempts to improve the bioavailability of hesperetin, such as the fabrication of hesperetin/hydroxypropyl-β-cyclodextrin complex nanoparticles using supercritical antisolvent technology [14], the preparation of nanophytosomes [15], and nanoemulsions [16].

The problem of poor biopharmaceutical properties attributed to poor solubility can be addressed by many technological approaches. One of them involves obtaining amorphous solid dispersions of active ingredients stabilized by a polymer matrix. Improved solubility and dissolution rates result from the breakdown of the compound’s crystal structure, which creates a supersaturated drug solution that increases bioavailability [17]. One promising technique that may be used to obtain amorphous solid dispersions is hot-melt extrusion. This process involves the thermal processing of a mixture of a pharmacologically active ingredient and a polymer, during which the polymer melts and disperses the active ingredient at the molecular level, thus achieving amorphous systems [18,19]. Hot-melt extrusion is a cost-effective process that offers a solvent-free approach and is easily scalable from the laboratory to the industrial level [18,19].

Considering the lack of research on formulating a combination product comprising curcumin and hesperetin, our studies aimed to utilize hot-melt extrusion for this purpose. First, we sought to evaluate the plasticizing effect of sweeteners on polyvinylpyrrolidone (povidone) PVP K30. Following the optimization of the first step, the second part focused on the preparation and characterization of a delivery system consisting of curcumin, hesperetin, PVP K30, and a plasticizer for potential combination therapy. This part involved three steps: (1) preparation of amorphous solid dispersions in various ratios by hot-melt extrusion, (2) solid-state characterization of amorphous solid dispersions, and (3) evaluation of in vitro biopharmaceutical properties such as dissolution rate, solubility, and permeability.

## 2. Materials and Methods

### 2.1. Materials

Curcumin (purity > 95%) was purchased from Xi’an Tian Guangyuan Biotech Co., Ltd. (Xi’an, Shaaxi Province, China), while hesperetin (purity > 95%) was obtained from Sigma-Aldrich (Sigma-Aldrich, St. Louis, MO, USA). Excipients were provided by the following manufacturers: PVP K30 by JRS Pharma (Rosenberg, Germany), erythritol by Intenson Europe (Karczew, Poland), xylitol by Santini (Poznań, Poland), and sorbitol by Biogo (Wrocław, Poland). Other reagents were as follows: dimethyl sulfoxide (DMSO, POCH, Gliwice, Poland), sodium hydroxide (Avantor Performance Materials Poland S.A., Gliwice, Poland), acetic acid 98–100% (POCH, Gliwice, Poland), sodium dihydrogen phosphate (PanReac AppliChem ITW Reagents, Darmstadt, Germany), and HPLC-grade methanol (J. T. Baker, Center Valley, PA, USA). High-quality, laboratory-grade pure water was prepared using a Direct-Q 3 UV purification system (Millipore, Molsheim, France; model Exil SA 67120). Prisma HT, GIT/BBB lipid solution, and acceptor sink buffer were supplied by Pion Inc. (Forest Row, East Sussex, UK).

### 2.2. Methods

#### 2.2.1. Preparation of Polymer–Plasticizer Mixtures

Polymer–plasticizer blends were prepared by dissolving appropriate amounts of ingredients needed to obtain 10 g of blend in 100 mL of purified water. To determine the plasticizing effect, blends with 5, 10, and 15% (*w*/*w*) sweeteners were prepared. Based on the determined equations of the linear function, the amount of plasticizer needed to obtain a polymer–plasticizer blend with a glass transition value of 120 °C was calculated. To prepare 10 g of the polymer–plasticizer mixture with a glass transition temperature of 120 °C, the following amounts were used: for erythritol mix 1.142 g of sweetener and 8.858 g of PVP K30; for xylitol mix 1.209 g of sweetener and 8.791 g of PVP K30; for sorbitol mix 1.338 g of sweetener and 8.662 g of PVP K30 were weighed, then transferred to a beaker and dissolved in 100 mL of purified water, stirring on a magnetic stirrer until a clear solution was obtained, then the solution was lyophilized (LyoQuest-85, Telstar, Terrassa, Spain) to obtain a powder. The freeze-drying process was carried out at a temperature of −82.1 °C and under vacuum at a pressure of 0.412 mbar. The freeze-dried products were powdered using a mill (Tube Mill Control Mixer, IKA, Warsaw, Poland). 

#### 2.2.2. Preparation of Hot-Melt Extruded Systems

**Figure 1:**
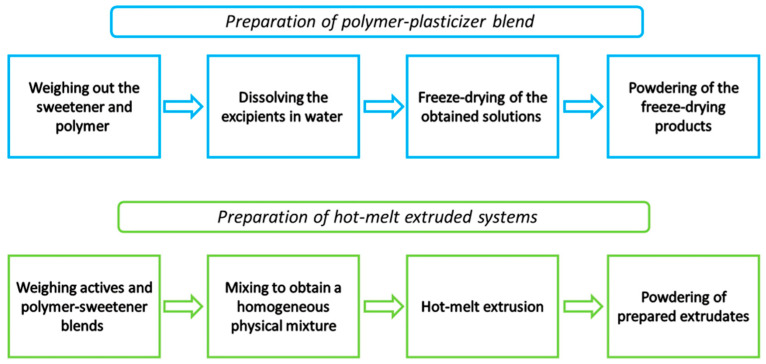
Scheme illustrating the preparation of polymer-platicizer blends and hot-melt extruded systems.

The hot-melt extrusion process was carried out in a HAAKE MiniCTW micro-conical twin screw extruder (Thermo Scientific, Karlsruhe, Germany). Components of systems were mixed using a mill (Tube Mill Control Mixer, IKA, Warsaw, Poland). Mixtures with 15 and 30% total content of active compounds were obtained. Then, the physical mixtures were fed manually into the hopper of the extruder at a barrel temperature of 150 °C and a screw speed of 90 rpm. The prepared extrudates were powdered by milling (Tube Mill control Mixer, IKA, Warsaw, Poland) and used for further studies. Figure 1 illustrates the preparation of the amorphous solid dispersions.

#### 2.2.3. X-ray Powder Diffraction (XRPD)

Diffraction patterns of the samples were obtained by X-ray diffraction (XRD, Panalytical Empyrean, Almelo, The Netherlands) equipment with the copper anode (CuKα—1.54 Å, 45 kV and 40 mA) using the Bragg-Brentano reflection mode configuration. The measurement parameters were set up to 5–60° 2theta with a 45 s step per 0.05°.

#### 2.2.4. Differential Scanning Calorimetry (DSC)

Thermal analysis was performed using a DSC 214 Polyma differential scanning calorimeter (Netzsch, Selb, Germany). Samples of about 5–10 mg were placed in crimped aluminum pans with a small hole in the lid. First, the samples were heated up to 80 °C and kept at this temperature for 8 min to remove water from the samples, then they were cooled down to 25 °C and heated again to 280 °C. To measure the glass transition temperature (Tg) of raw compounds, they were heated to 280 °C, then cooled down and heated again to 280 °C. The measurements were performed at a heating rate of 20 °C/min and a cooling rate of 10 °C/min under a nitrogen atmosphere with a flow rate of 30 mL/min. The Tg was taken as a midpoint between onset and endpoint temperatures.

In the examination of Tgs values of PVP K30–sweetener mixtures, a heating–cooling–heating cycle was used. The first heating aimed to eliminate residual water. The sample was heated to 200 °C at a heating rate of 20 °C/min, then cooled down to 25 °C at 10 °C/min, and heated again to 200 °C at a heating rate of 20 °C/min.

To determine the glass-forming ability, different heating–cooling–heating cycles were performed. First, about 5–10 mg of the sample was heated about 10 °C above the melting point of the compound, then cooled to −65 °C at different rates (40, 20, 10, and 5 °C/min) followed by a second heating up to 10 °C above the melting point at 20 °C/min.

#### 2.2.5. Fourier-Transform Infrared Spectroscopy (FTIR-ATR)

FTIR-ATR spectra were measured between 400 cm^−1^ and 4000 cm^−1^, with a resolution of 1 cm^−1^, with a Shimadzu IRTracer-100 spectrometer equipped with a QATR-10 single bounce, a diamond extended range, and LabSolutions IR software (version, 1.86 SP2, Warsaw, Poland). Amorphous forms of compounds were prepared by DSC as described in the differential scanning calorimetry section above.

#### 2.2.6. Physical Stability

The physical stability was observed for up to 6 months at 25 ± 0.2 °C and 50 ± 0.2 °C in dry conditions provided by silica gel. The samples were taken out after 1, 2, 3, and 6 months and examined for the presence of crystallinity by XRPD. The samples were stored in a light-protected condition in glass vials.

#### 2.2.7. High-Performance Liquid Chromatography (HPLC) Analysis

Concentrations of curcumin and hesperetin in samples collected during solubility, dissolution, and permeability studies were determined using HPLC with the DAD detector (HPLC-DAD). Shimadzu Nexera (Shimadzu Corp., Kyoto, Japan) equipped with an SCL-40 system controller, a DGU-403 degassing unit, an LC-40B XR solvent delivery module, a SIL-40C autosampler, a CTO-40C column oven, and an SPD-M40 photodiode array detector were employed in this investigation. For the stationary phase, a Dr. Maisch ReproSil-Pur Basic-C18 100 Å column with 5 µm particle size and 250 × 4.60 mm (Dr. Maisch, Ammerbuch-Entringen, Germany) was used. The mobile phase was HPLC-grade methanol:0.1% acetic acid (80:20 *v*/*v*). The mobile phase was vacuum filtered through a 0.45 µm nylon filter (Phenomenex, Torrance, CA, USA). The experimental conditions were as follows: a 1.0 mL/min flow rate, a wavelength of 420 nm for curcumin and 288 nm for hesperetin, and a column temperature of 30 °C. The injection volume differed depending on the assay. For the solubility study, it was 2 µL, whereas for the dissolution and permeability assays, it was 10 µL. The duration of the run was 10 min. The retention time was 6.644 min for curcumin and 4.221 min for hesperetin. Method validation parameters (Table S1) and chromatograms (Figure S1) were placed in the Supplementary Materials.

#### 2.2.8. Preparation of Media for Solubility Studies and Dissolution

Phosphate buffer at pH 6.8 was prepared as follows: 250 mL of 0.2 N potassium dihydrogen phosphate solution was placed in a 1000 mL volumetric flask, and then 112 mL of 0.2 N sodium hydroxide solution was added. The volume was made up to 1000 mL with purified water. Hydrochloric acid solution pH 1.2 was prepared from the analytically weighed amount following the manufacturer’s (Alfachem, Poznań, Poland) recommendations.

#### 2.2.9. Solubility Studies

An excess amount of powder (corresponding to 6 mg of each active substance) was placed in a 10 mL glass tube. Then, 2.0 mL of phosphate buffer (pH 6.8) or HCl solution (pH 1.2) was added and stirred at 100 rpm at room temperature (25 ± 0.1 °C) for 24 h. The obtained solutions were diluted 1:20 *v*/*v* with water, filtered through a 0.2 μm PTFE membrane filter (Sigma-Aldrich, St. Louis, MO, USA), and analyzed using the HPLC method described above.

#### 2.2.10. Dissolution Studies

The dissolution study was performed using the paddle apparatus (Agilent 708-DS dissolution apparatus, Santa Clara, CA, USA). The amount of the compound and extruded systems corresponding to 5 mg of each plant-origin active ingredient was added to a gelatin capsule (size: 1), placed in a spring used as a sinker, and then added to the dissolution medium. The vessels were filled with 500 mL of phosphate buffer pH 6.8 or HCl pH 1.2; the temperature was maintained at 37 ± 0.1 °C, and the paddle speed was set to 50 rpm. Aliquots of 2.0 mL liquid were taken out with the replacement of equal volumes of temperature-equilibrated media at the following time intervals: 5 min, 15 min, 30 min, 1 h, 2 h, 3 h, 4 h, 5 h, and 6 h, then samples were filtered through a 0.2 μm PTFE membrane filter and analyzed by HPLC. Each sample was tested 3 times.

#### 2.2.11. Permeability Studies

In vitro, gastrointestinal (GIT) and blood–brain barrier (BBB) permeability was studied using the Parallel Artificial Membrane Permeability Assay (PAMPA) models. The sandwich is composed of two 96-well microfilter plates. The PAMPA systems contained two chambers: the donor chamber at the bottom and the acceptor chamber at the top. The chambers were separated by a 120 μm thick PVDF membrane coated with a 20% (*w*/*v*) dodecane solution of a lecithin mixture (Pion, Inc., Forest Row, East Sussex, UK). The donor and acceptor solutions were provided by the manufacturer. The donor solution was diluted according to the manufacturer’s recommendations and adjusted to a pH of ≈6.8 for the GIT assay and to a pH of ≈7.4 for the BBB assay using 0.5 M NaOH. The plates were combined and then incubated for 4 h for both models in a humidity-saturated atmosphere with the temperature set to 37 °C. The samples for the donor compartments were first prepared in the same manner as for the solubility studies, using purified water. Then, the systems were diluted 1:5 *v*/*v* with water, filtered through a 0.2 μm PTFE membrane filter, and further diluted 1:1 *v*/*v* with DMSO. Next, the obtained solutions were diluted 1:1 *v*/*v* with the donor solution for the GIT and BBB assays and placed in the donor compartments. The results are expressed as a concentration of the compound in the acceptor solution.

#### 2.2.12. Statistical Analysis

Results are expressed as the mean ± standard deviation. Statistical tests were performed using a one-way analysis of variance (ANOVA), and statistical differences using Duncan’s tests with a significance threshold of *p* < 0.05 were determined. All statistical analyses were performed using Statistica 13.1 software (TIBCO Software Inc., Palo Alto, CA, USA).

## 3. Results and Discussion

In this study, we focused on the manufacture of amorphous solid dispersions comprising curcumin and hesperetin in the polymer matrix to develop a fixed-dose system for the potential combination therapy using both plant-derived compounds. The motivation for this approach was the poor solubility of the compounds, which hampers their pharmacological properties. Also, the study by Lee et al. suggested the potential benefits of co-administration of both compounds [20]. In their study, the authors attempted to determine whether the simultaneous administration of curcumin and hesperetin has promising neuroprotective properties. The authors indicated that co-administration of these two compounds made the therapy more comprehensive, acted on more molecular targets, and showed better results in comparison to the compounds used separately [20]. Although they did not explicitly conclude that there was synergism of action between the compounds, it might be beneficial, due to the said advantages, to develop a fixed-dose combination system with better pharmaceutical properties.

Based on a literature review, we concluded that PVP K30 (also called Kollidon 30) might be the best carrier for curcumin dispersion. Nogami et al. found that PVP significantly increased the solubility of curcumin, which the authors attributed to specific interactions between the pyrrolidone backbone and curcumin, promoting inhibition of crystallization and maintenance of the supersaturated state. Hence, we directed our efforts to use this polymer [21].

In the first stage of this study, a feasibility study was carried out to check if PVP K30 can be used in hot-melt extrusion. Based on the literature, it is evident that using PVP K30 in hot-melt extrusion is a challenge. Pina et al. attempted to extrude olanzapine with PVP K30 [22]. The processing temperature was 160 °C, which is close to the glass transition temperature (Tg) of PVP K30. The extrusion of the mixture with a drug load of 50% was partially successful. The authors anticipated that the drug would act as a plasticizer, which would improve extrudability. It was concluded that the process was difficult but possible. However, the extrudates had a block-like shape with a rough appearance instead of being thin spaghetti-like strands. By the appearance of the extrudate, it can be assumed that the process did not run smoothly, and therefore the process conditions were not optimal, which is related to the unsuitable process temperature. Nevertheless, the resulting formulation was amorphous [22]. Xi et al. attempted to obtain an amorphous solid dispersion of lacidipine with various polymers via hot-melt extrusion [23]. One of the polymers was PVP K30, but it was excluded from further studies due to its poorer impact on dissolution rate in comparison to the other carriers. The reason for this observation could be that the drug was not sufficiently dispersed in the carrier. This may have occurred due to an unsuitable process temperature of 175 °C, at which the polymer may not have sufficiently low viscosity, allowing the drug to disperse at the molecular level in the polymer matrix [23].

Based on the literature, processing curcumin via hot-melt extrusion is a challenging task. Fan et al. indicated that curcumin extrudates obtained by the process at 160 °C showed a lower content of the active compound, which the authors attributed to partial thermal degradation under processing conditions. On the other hand, when the process was carried out at 140 °C, the presence of curcumin in partially crystalline form was found [24]. Based on the above, we determined that extrusion should be carried out at 150 °C. Bearing in mind the aforementioned findings, it is impossible to extrude curcumin using PVP K30 on its own, as the polymer shows a Tg of about 168 °C. The reason is that efficient extrusion requires that the processing temperature be at least 20 °C higher than the Tg of the extruded blend, which mainly depends on the carrier used [25,26,27].

The difficulties in obtaining amorphous solid dispersions of hesperetin using hot melt extrusion have not been described so far, and no attention has been paid to the potential thermal decomposition at temperatures below 200 °C; hence, we considered that this compound would be stable during the process and would not be as technologically demanding as curcumin.

Facing formulation difficulties with PVP K30, we decided to use a plasticizer to improve the extrudability, effectively lowering the Tg of the polymer and ensuring its good processability at temperatures below the Tg. Sugar alcohols such as erythritol, sorbitol, and xylitol are widely used in the food and pharmaceutical industries. They can be substitutes for confectioners’ sugar but also act as excipients in medicinal products [28,29]. Several papers have reported on the use of sweeteners as plasticizers useful in the production of various films [30,31,32]. Moreover, attempts are being made to use them effectively in formulations to improve solubility. Xylitol has been used as a co-crystal co-former to enhance the dissolution rate of felodipine [33].

**Figure 2:**
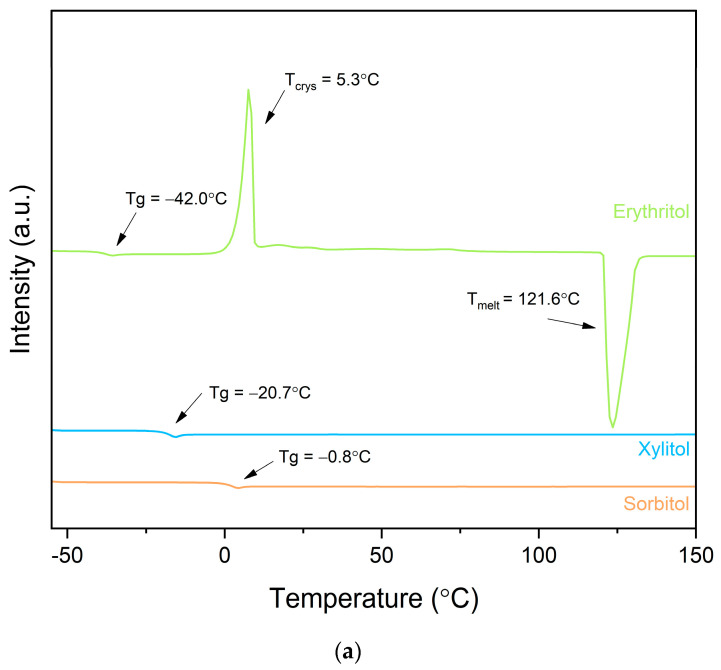

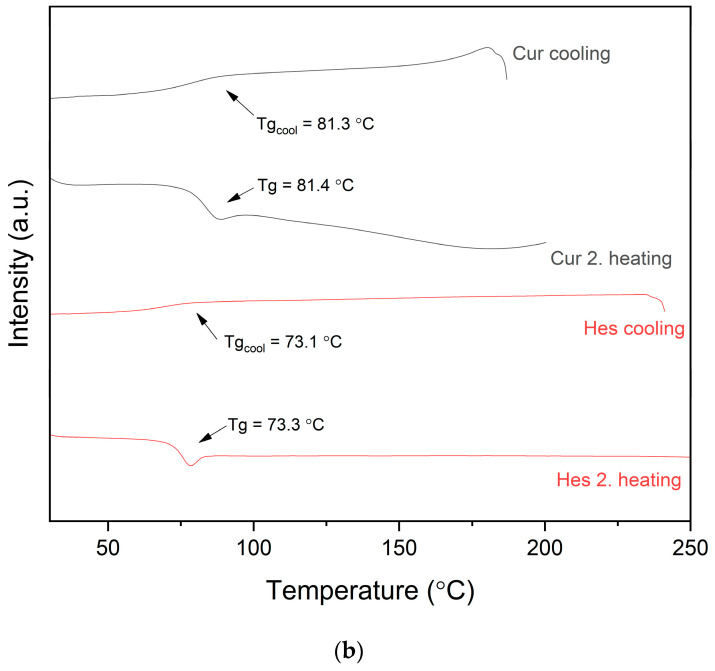
Glass-forming ability assessment of sweeteners (**a**) and active compounds (**b**). For sweeteners, a second heating run is shown, whereas for active compounds, both cooling and second heatings are shown. Arrows point to thermal events.

First, we decided to determine the glass-forming ability of sweeteners, which provided us with information about the ease of obtaining the amorphous state. The ease of forming the amorphous state makes it less likely that compound crystallization occurs quickly, which could also promote the crystallization of the active compounds. In assessing the glass-forming ability, we used the approach described by Alhalaweh et al. [34]. We employed several cooling rates (20, 10, and 5 °C/min), based on the findings of Blaabjerg et al. [35]. As one can see, xylitol and sorbitol can be classified as good glass formers since they did not crystallize in the heating–cooling–heating cycles used, even when the cooling rate was as low as 5 °C/min, presenting Tgs of −20.7 °C and −0.8 °C, respectively. Therefore, they can be classified as Class III glass formers. On the other hand, we were not able to obtain a thermogram of erythritol without the occurrence of crystallization; even a high cooling rate of 40 °C/min followed by fast heating of 40 °C/min and Tg at −42.0 °C was seen, followed by crystallization at 5.3 °C and then melting at 121.6 °C. These events were observed in the second heating run (Figure 2a); hence, erythritol is a Class II glass former. Talja and Roos et al. [36] found that erythritol rapidly crystallizes from its melt, which confirms our observation. Therefore, it can be expected that erythritol can easily crystallize from the dispersion due to its high tendency to crystallize.

Prior to the development of the initial amorphous solid dispersions, we also estimated the glass-forming abilities of curcumin and hesperetin (Figure 2b) to ensure that the formulation approach using amorphous compounds was reasonable. The procedure was carried out as for sweeteners, applying different cooling rates (20, 10, and 5 °C/min). We found that both active compounds show good glass-forming abilities (Class III glass formers) since crystallization was not observed in the cooling and second heating runs.

To obtain the polymer–plasticizer blends, we first obtained a polymer–plasticizer solution in water, which ensured that the polymer and plasticizer could interact more effectively and had a better opportunity of mixing. These solutions were then freeze-dried to form powders capable of further processing by hot-melt extrusion.

**Figure 3:**
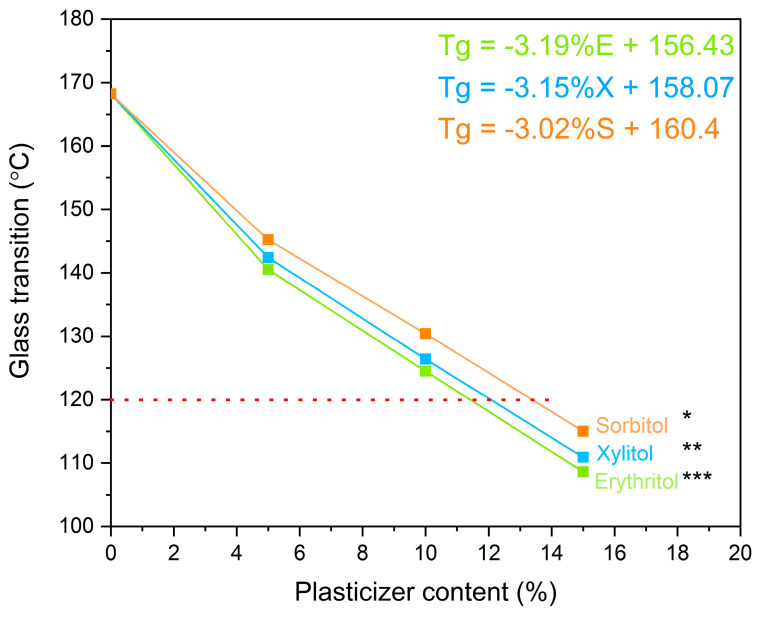
The dependence of the sweetener’s content on the Tg value of the PVP K30–sweetener blend. The red dashed line shows the target Tg. Asterisks indicate statistical difference (*p* < 0.05).

The sweeteners (erythritol, xylitol, and sorbitol) exhibited a plasticizing effect, effectively reducing the Tg of the polymer. To determine their plasticizing potential, blends with different contents of each sweetener (5%, 10%, and 15%) were prepared. Figure 3 presents the Tg-compositional dependence for each polymer/sweetener combination. As can be seen, erythritol proved to be the most effective plasticizer, while sorbitol was the weakest among those tested. This may be important for the performance of amorphous solid dispersion, as the polymer is an ingredient that inhibits crystallization and maintains the supersaturated state [37]. Choosing erythritol as the plasticizer can ensure a higher proportion of polymer in the dispersion while achieving the desired thermal properties.

The dependence of the plasticizer content on the Tg (Figure 3) was found to be linear for concentrations ranging from 5 to 15% of the plasticizer content; thus, it was possible to accurately determine the composition of the polymer–plasticizer blend with a specific Tg value from the equation of the straight line. Erythritol being the most effective plasticizer can be explained when the glass transition values of amorphous forms of sweeteners are taken into consideration. We showed that the glassy transitions for erythritol, xylitol, and sorbitol are −42.0 °C, −20.7 °C, and −0.8 °C, respectively. Furthermore, depending on the proportions of the two components in the mixture, the value of the mixture’s glass transition will lie between the values of the glass transitions of the two components when they form a homogeneous mixture [38]. Given that amorphous erythritol has the lowest glass transition value, its plasticizing strength will be most pronounced. Moreover, it is worth noting that the glassy transitions of pure sweeteners are arranged in the order erythritol < xylitol < sorbitol, where erythritol shows the lowest value. We can represent the same order for the plasticizing strength of sweeteners. At equal percentages of sweeteners in the blend with PVP K30, the blend with erythritol demonstrated the lowest Tg value.

**Figure 4:**
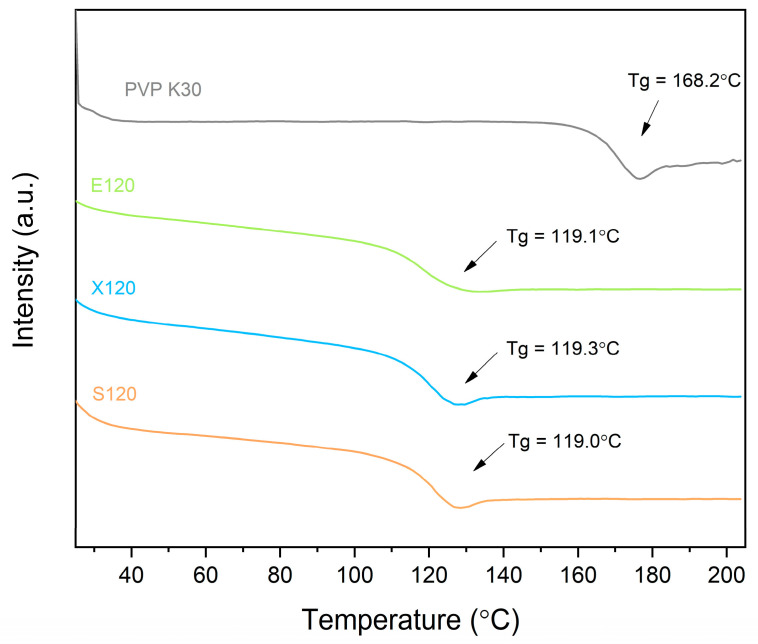
DSC showing Tg values of the designed polymer–plasticizer mixture. Arrows point to Tg values.

After confirming that sweeteners can act as plasticizers, we proceeded with the preparation of the polymeric matrix suitable for extrusion. The hot-melt extrusion process involves processing a polymeric material above its Tg [39]. Hence, the efficiency of the hot melt extrusion process will mainly depend on the processability of the polymer–plasticizer blend. The design of the final polymer–plasticizer mixture could go two ways. It can be assumed that all blends would have the same plasticizer content, for example, 10%, or that the blends would have the same Tg value, which is possible to determine based on the data presented in Figure 3. It was decided to prepare the blends so that they would have the same Tg of 120 °C. This approach ensured that we could run the process smoothly without modifying it based on the Tg of individual blends. As mentioned earlier, it was decided that extrusion would be carried out at 150 °C; hence, the temperature of Tg = 120 °C should allow an efficient process and provide sufficient physical stability for the resulting amorphous solid dispersion. The DSC thermograms of the designed polymer–plasticizer blend are shown in Figure 4.

Having completed the studies on polymer–plasticizer mixtures, the next step was to obtain amorphous solid dispersions containing the active compounds via hot-melt extrusion. The quaternary systems (polymer–plasticizer–curcumin–hesperetin) were prepared at two different mass ratios of the active ingredients, 30 and 15% (curcumin:hesperetin ratio 1:1 *w*/*w*). The following notation is used throughout the text: the capital letter denotes the sweetener used (E = erythritol, X = xylitol, and S = sorbitol), while the number denotes the total content of active compounds (30 = 30% total content of curcumin and hesperetin in the system; 15 = 15% total content of curcumin and hesperetin in the system).

**Figure 5:**
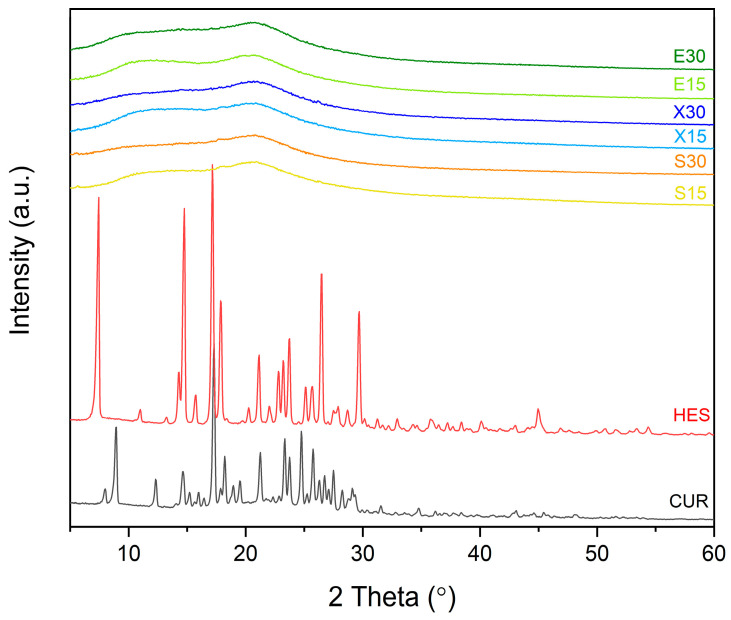
XRPD analysis of curcumin and hesperetin “as obtained” powders as well as of the extruded amorphous solid dispersions.

Figure 5 displays the diffractograms of raw plant-origin active compounds and the extruded systems. Raw curcumin presented sharp peaks at 8.85°, 17.24°, 21.16°, 23.27°, 24.67°, 24.68°, 25.63° 2theta, whereas raw hesperetin presented peaks at 7.33°, 14.64°, 17.10°, 17.82°, 21.08°, 26.39°, 29.63° 2theta. A “halo effect” in the XRPD patterns of the obtained dispersions indicated that they were amorphous. Therefore, hot melt extrusion successfully allowed the production of amorphous solid dispersions.

**Figure 6:**
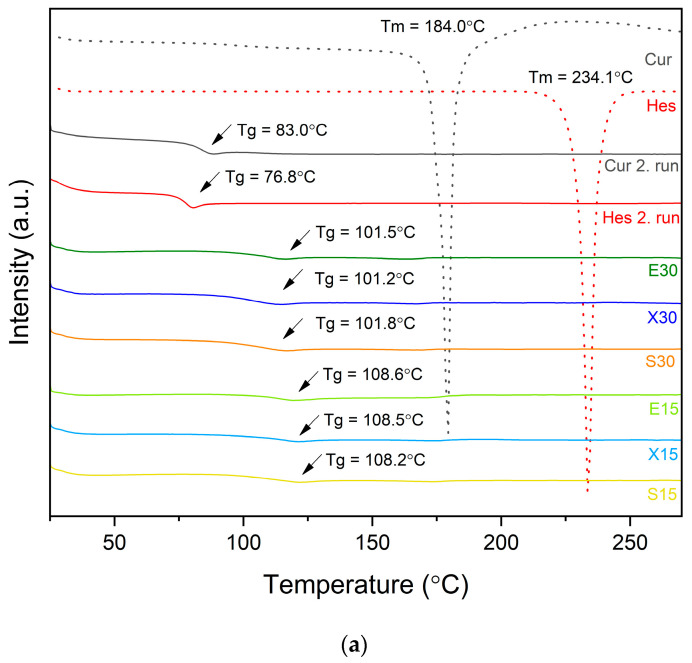

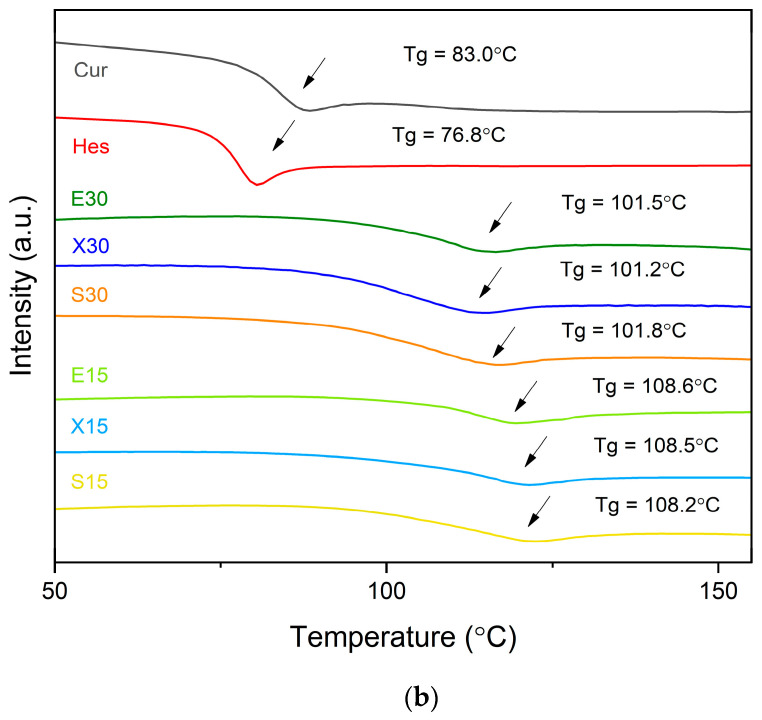
DSC thermograms of raw compounds and obtained dispersions (**a**). Tg values of amorphous systems (**b**). Arrows point at Tg values.

The DSC thermograms (Figure 6) of amorphous solid dispersions showed a single Tg, suggesting that active compounds are molecularly dispersed in the polymeric carriers and confirming the good miscibility of dispersions components [40]. Additionally, the disappearance of endothermic peaks corresponding to melting temperatures of compounds is in agreement with XRPD results and confirms the amorphous nature of the systems, since no sign of crystallinity was detected by this analysis. The active compounds, curcumin and hesperetin, have lower Tg values (Tg = 83.0 °C and Tg = 76.8 °C, respectively) than the binary carrier. Therefore, they will decrease the Tg of the carrier, acting as plasticizers. Therefore, carriers with a higher Tg will exhibit an anti-plasticizing effect on active compounds; hence, they will hamper their molecular mobility, ensuring the stability of the amorphous form of the compounds [41]. Amorphous solid dispersions revealed lower Tg values than carriers, but higher than those of pure amorphous compounds. In addition, dispersions with 30% of active compounds exhibited lower Tg values than dispersions with 15%, which confirms the plasticizing property of compounds. The intermediate value of Tg can be explained by the Gordon–Taylor equation. As it is known, the mixing of two components with different Tg, assuming the miscibility of the components, should lead to a single-phase system with a Tg temperature value within the range of Tg of the individual components, depending on the percentage of components [38]. This principle is applicable in the case of obtained amorphous solid dispersions since Tg values lie between Tgs of dispersions’ constituents.

**Figure 7:**
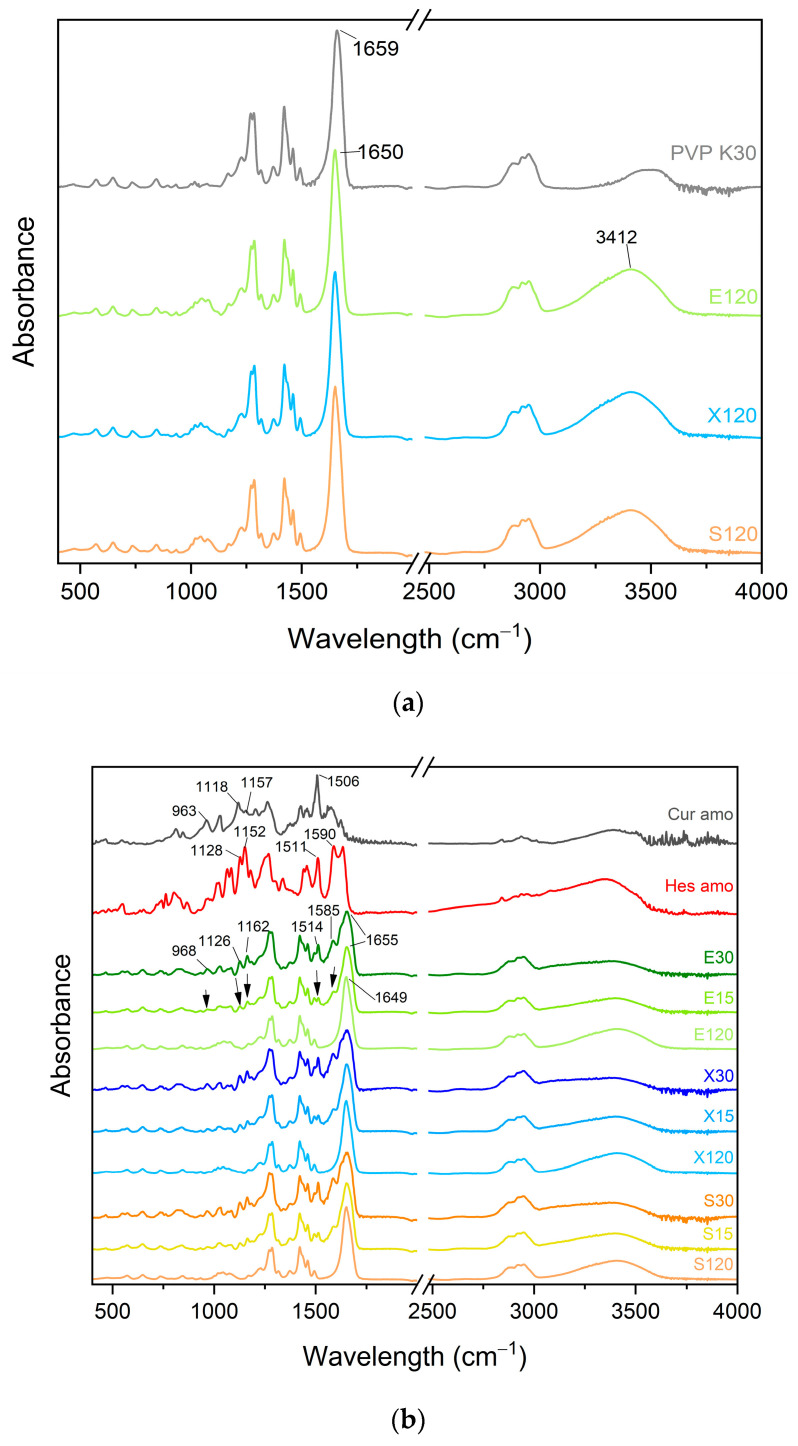
FTIR-ATR spectra of PVP K30 and modified polymer (polymer-plasticizer mixture) (**a**) as well as amorphous raw compounds, carriers, and amorphous solid dispersions (**b**). Arrows point at changes.

FT-IR spectra of the various systems investigated are presented in Figure 7. The differences between the crystalline and amorphous spectra of curcumin and hesperetin have already been described [42,43].

Pure PVP K30 can only act as a hydrogen bond acceptor due to the presence of a carbonyl group in the pyrrolidone ring [44,45]. The addition of a sweetener provides more opportunities for interactions to occur due to the presence of multiple hydroxyl groups, which gives the binary carrier the ability to act as a hydrogen bond donor and/or acceptor. This creates new possibilities for stabilizing the amorphous state.

The spectra of PVP K30 and the individual sweeteners are very similar (Figure 7a). The spectra of the modified carriers are dominated by the bands of PVP K30, but some changes can be observed, like the appearance of a more intense region around 3400 cm^−1^, which confirms the presence of hydroxyl groups. In addition, the carbonyl peak of PVP K30 shifted from 1659 cm^−1^ to 1650 cm^−1^. The observed changes suggest the incorporation of sweeteners between the polymer chains and the presence of PVP K30–sweetener interactions. The embedding of the sweetener molecules between the polymer chains may have weakened the interactions in PVP K30 itself between the chains, which reduced the Tg values and improved the processability in hot-melt extrusion.

The FT-IR spectra of the extruded systems are similar to each other (Figure 7b). It can be observed that there was a band shift of the carrier from 1649 cm^−1^ to 1655 cm^−1^. The band of the system at 1585 cm^−1^ may be a shifted band of hesperetin from 1590 cm^−1^, while the band at 1514 cm^−1^ may be a shifted band of curcumin from 1506 cm^−1^ or a slightly shifted band of hesperetin at 1511 cm^−1^. In addition, the band of the system at 1162 cm^−1^ can be a moved band of curcumin at 1157 cm^−1^ or hesperetin at 1152 cm^−1^. The band at 1126 cm^−1^ of the system can be an altered band of curcumin at 1118 cm^−1^ or hesperetin at 1128 cm^−1^. The band at 968 cm^−1^ could be the original curcumin band at 963 cm^−1^. The systems were compared to an amorphous form of active compounds and a polymer-plasticizer blend. The appearance of the spectra suggests that both curcumin and hesperetin, as well as the carriers, are involved in interactions.

The addition of sweeteners enabled not only processability but also had an impact on the stability of the systems. The sweeteners used belong to the group of alcohol sugars; they have numerous hydroxyl groups in their chemical structure that can participate in the formation of hydrogen bonds. Additional enrichment of the carrier with functional groups that can participate in intermolecular interactions can lead to additional stabilization of molecularly dispersed active compounds. API-polymer interactions will additionally reduce molecular mobility, leading to greater physical stability [46]. Based on the results of the FT-IR/ATR analysis of the prepared dispersions, we expect that, in addition to the steric hindrance due to the polymer chains, plant-active compound–polymer interactions may considerably contribute to physical stability.

**Figure 8:**
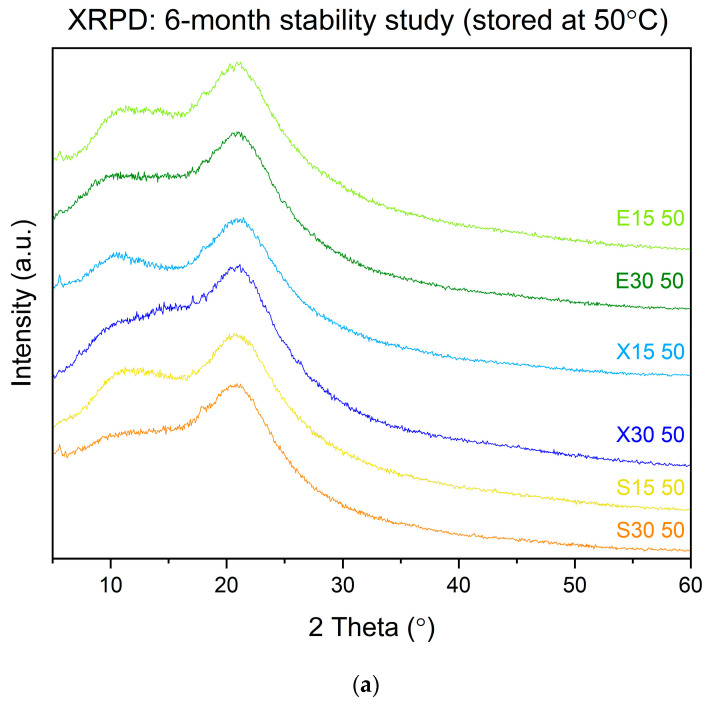

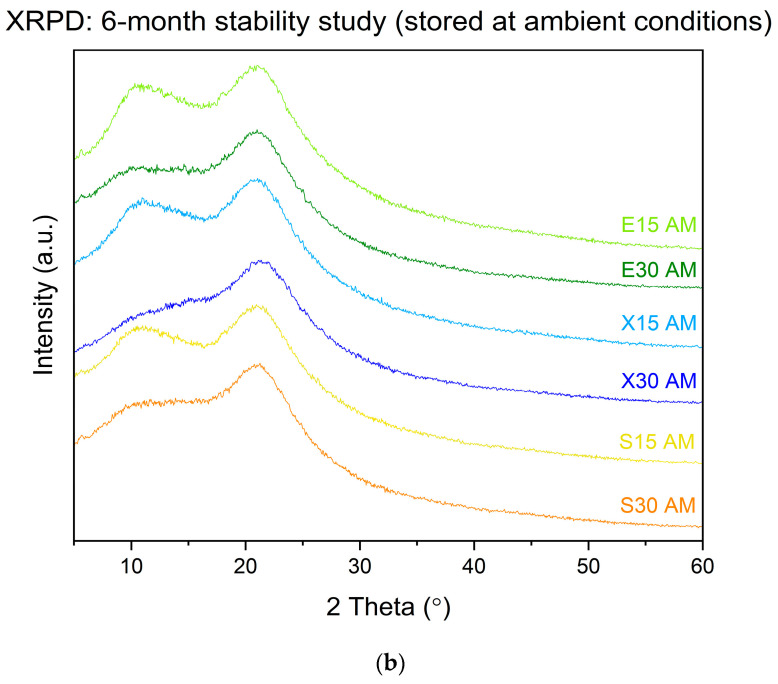
XRPD results of physical stability studies after 6 months of storage at 50 °C (**a**) and at ambient conditions (**b**).

When developing amorphous systems, physical stability must be taken into account. From the shelf-life point of view, it is best that formulations are stored at room temperature, and this should apply to formulations comprising amorphous solid dispersions. The extruded systems were initially kept in two different environmental settings: ambient (also referred to as shelf conditions) and at a higher temperature (50 °C) without the humidity factor. The XRPD investigation showed that the systems were stable at both storage conditions after 6 months since XRPD patterns did not display any peaks (Figure 8). Interestingly, dispersions where erythritol was used as a carrier presented an amorphous pattern after 6 months. We previously showed that this sweetener has a high tendency to crystallize (Figure 1); however, crystallization of this compound was not detected. This may be due to molecular interactions contributing to the stabilization of the amorphous form of erythritol in the dispersions, thus preventing recrystallization. The polymer exhibited an anti-plasticizer effect that resulted in a considerable increase in the Tg and therefore in a restriction of global molecular mobility (α-relaxation), mainly responsible for crystallization onset [47]. As a result, crystal formation and growth were prevented even with erythritol. With the Tg values above 100 °C and applying the so-called “Tg-50 rule” [48], it can be concluded that we can expect long-term physical stability for the extruded systems.

The next step of this study, after confirming the amorphization and interactions in the systems, was to determine pharmaceutical parameters such as dissolution rate, solubility, and permeability.

Considering the solubility test (Table 1), the greatest solubility was achieved under pH 6.8 buffer conditions for amorphous solid dispersions containing 15% of active compounds. The improvement was 42,386-fold, 42,343-fold, and 42,286-fold for curcumin and 1184-fold, 1182-fold, and 1181-fold for hesperetin for the E15, X15, and S15 systems, respectively. The very significant improvement in apparent solubility values could be due to the amorphous nature of the active compounds and the presence of sugar alcohols acting as hydrotropic agents. It is known that polyols can increase the solubility of many poorly soluble compounds [49]. The lower values measured in HCl solutions are consistent with previous observations made by Paulazzi et al. for curcumin [50] and Lucas-Abellan et al. for hesperetin [51].

**Figure 9:**
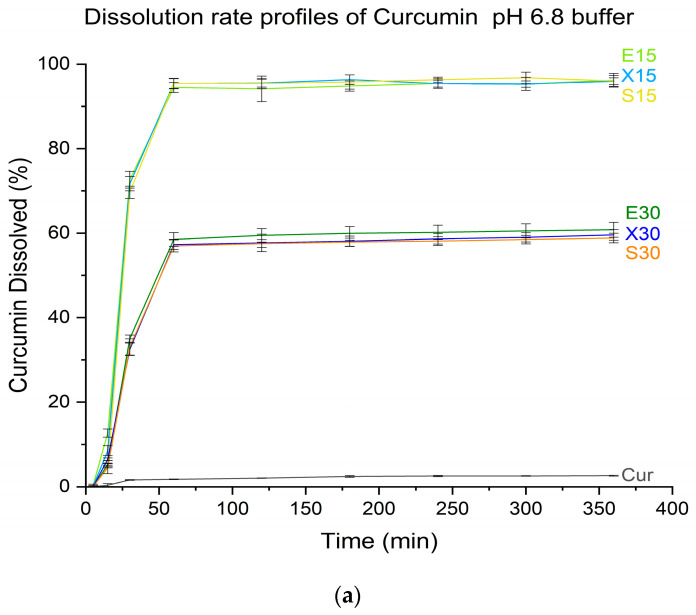

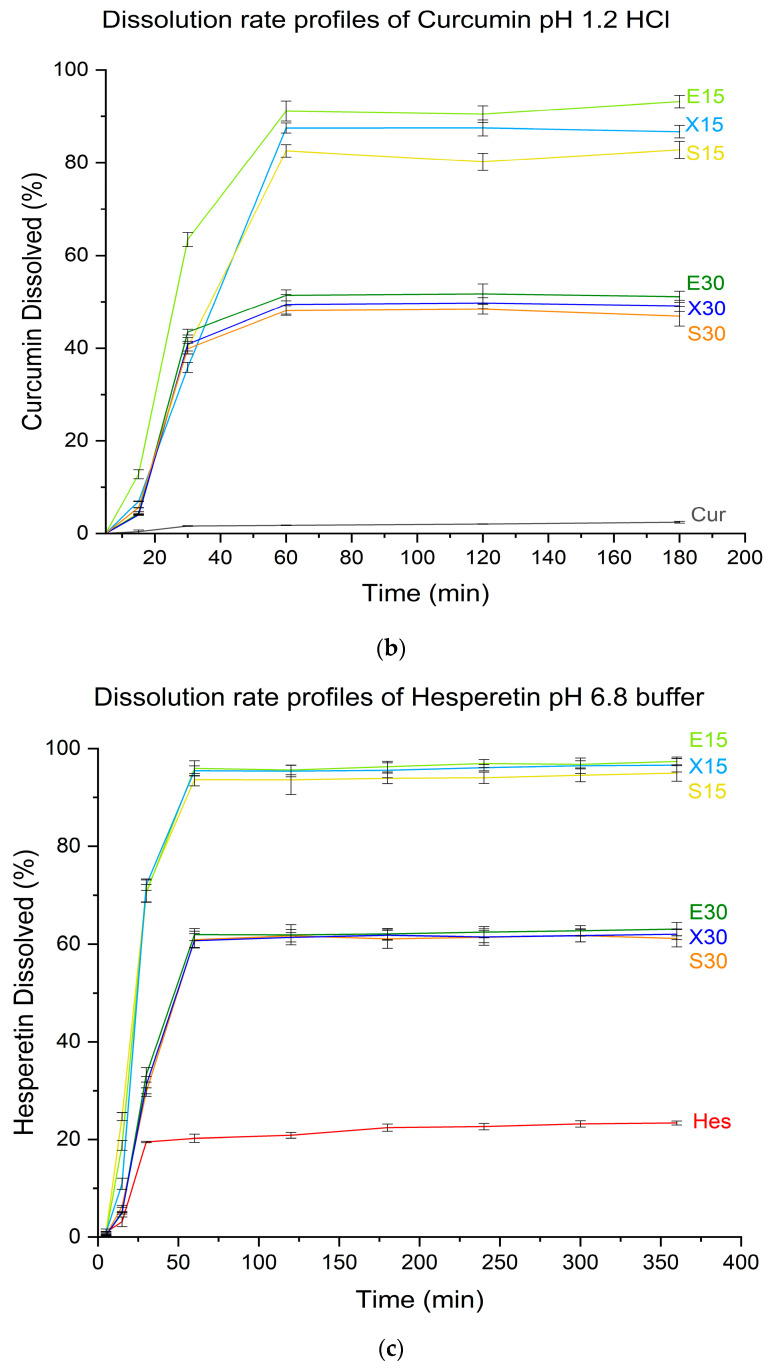

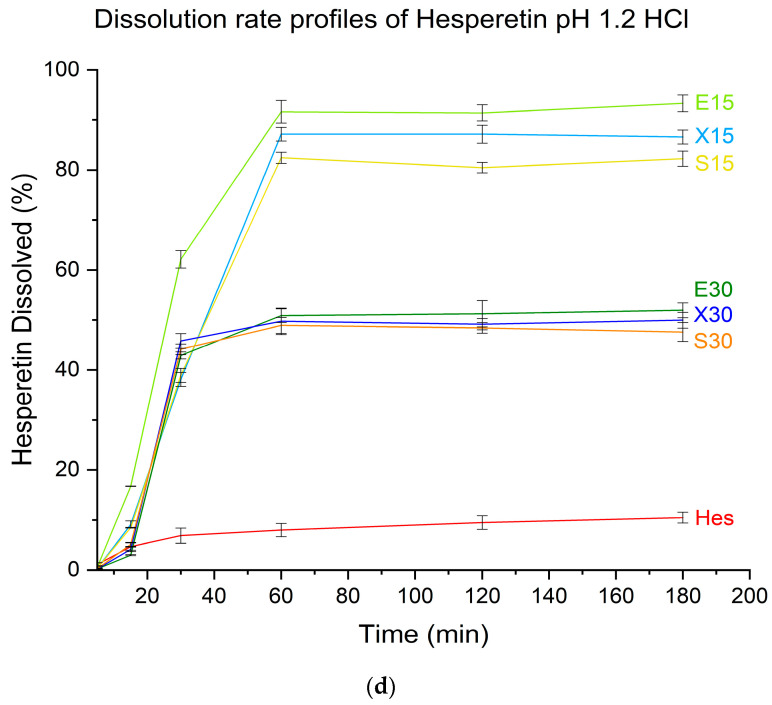
Dissolution rate profiles for amorphous systems of curcumin in pH 6.8 buffer (**a**), pH 1.2 HCl (**b**), and hesperetin in pH 6.8 buffer (**c**), pH 1.2 HCl (**d**).

Considering dissolution studies, amorphous solid dispersions resulted in a supersaturated state (Figure 9), which means higher amounts of the compounds dissolved than expected compared to the crystalline solubility (Table 1). In the case of curcumin, there was a 38-fold improvement in solubility for systems E15 and a 23-fold improvement for E30, while for hesperetin, it was 4- and 3-fold, respectively, compared to pure compounds. The dissolution rate profiles for E15, X15, and S15 systems are similar in pH 6.8 buffer. It can be concluded that in this case, the amount of polymer had a lesser impact on the performance of the systems, and it was sufficient to maintain the supersaturated state. There is less polymer in S15 systems compared to E15 and X15 due to the weaker plasticizing effect of sorbitol. However, despite this, the release profile for this system was not worse in comparison to the others. It can be seen that the amount of polymer affects the resulting dissolution rate profile and apparent solubility. Systems with more polymers—a lower drug load—performed better. They showed a better improvement in solubility and dissolution rate. When tested in pH 6.8 buffer, systems with 15% of active compounds presented similarly; they allowed almost complete dissolution of active compounds. On the other hand, for pH 1.2 HCl conditions, differences in behavior can be seen. The best improvement in apparent solubility was observed for E15 systems, which enabled the dissolution of a similar amount of active compounds as in pH 6.8 buffer. X15 and S15 systems performed worse in pH 1.2 HCl, reaching 84.5% and 78.2% of dissolved curcumin and 82.2% and 78.4% of dissolved hesperetin, respectively, while in pH 6.8 buffer, these systems allowed the dissolution of 95.4% of curcumin and 95.6% of hesperetin for X15 and 95.3% of curcumin and 95.4% of hesperetin for S15. It is important to note that we observed incomplete dissolution of the 30% systems with the particles of the system floating in the dissolution medium.

It is noteworthy that the dispersions helped to achieve a supersaturated state, which is a key factor for improving the overall bioavailability of active compounds from amorphous systems. We can observe the “spring and parachute” phenomenon. Fast dissolution of the tested amorphous solid dispersions created high drug concentrations, generating a supersaturating solution (spring). This state was maintained throughout the course of the dissolution study (parachute) [52]. The supersaturated state produced by the amorphous state is a driving force for enhanced absorption since it boosts passive diffusion [53]. Thus, the developed amorphous solid dispersions may favorably influence absorption and lead to increased blood concentrations of curcumin and hesperetin.

Although the solubility study showed a considerable improvement in solubility, which might have suggested that in the dissolution rate study, all amorphous systems should provide almost complete dissolution and release of compounds from the polymer matrix, this was not the case, and only systems of 15% content allowed dissolution of a given dose when tested in pH 6.8 phosphate buffer. These differences were exacerbated under pH 1.2 HCl conditions, where only the E15 system allowed the release of the loaded dose. The differences in the tests are due to their conditions, particularly the volume of the medium itself. In the dissolution rate study, the volume of the medium was 500 mL, while in the solubility study, it was 2 mL. The dissolved polymer increases the medium’s viscosity, which restricts molecular movement and therefore promotes a reduction in the likelihood of crystallization by making nucleation and crystal formation more difficult. In a denser medium, the impact of the medium’s properties will be more pronounced. Multiple polymer chains may surround a single molecule, reducing the tendency for drug-rich phase creation and crystallization to emerge. In the case of the dissolution study, the effect of the adherence of polymer chains to individual molecules will be seen to a lesser extent, which will result in a weakening of the polymer’s ability to prevent crystallization. Nevertheless, both studies provide essential information on the characteristics of amorphous solid dispersions and are important in amorphous solid dispersion development. Dissolution studies are used to simulate the behavior of dispersions more accurately in the body after administration, whereas solubility studies describe how much solubility improvement can be achieved and how high a concentration of active compounds can be reached.

Also, permeability is an important biopharmaceutical parameter affecting API bioavailability. For a drug to show action, it must first dissolve in the aqueous environment of the GI tract and then pass through the intestinal mucosa and into the bloodstream [54]. Absorption is a complex process and depends on many factors, such as the amount of API in the absorbable form, which affects not only the efficiency of passive diffusion but also the efflux of API molecules back into the intestinal lumen [54]. The advantage of the amorphous state is the high absorptive flux across the intestinal epithelium [55]. There is a supersaturated state and an increase in the free fraction of the drug that can be absorbed. This phenomenon is the driving force behind the absorption of active compounds [56]. The polymer prevents the crystallization of compounds in the amorphous state, extending the relevant absorption time frame. This results in better therapeutic outcomes. It has been shown that the supersaturation state contributes to better bioavailability because more of the active substance is present in the blood [57,58]. In addition, the supersaturation state generated by amorphization also makes it possible to bypass the negative effect of efflux on bioavailability. This is due to the saturation of proteins that actively transport APIs out of the cell, such as glycoprotein-P. These proteins have limited throw-out capacity. Exceeding the threshold of the glycoprotein-P transporter and its saturation greatly increases permeability. Such a situation is only possible with high levels of saturation, through which a higher fraction of the administered dose can escape the efflux transporter [59]. Curcumin and hesperetin are substrates of efflux proteins [60,61], so generating significant supersaturation is an opportunity for them to overcome the physiological barriers, which will improve their bioavailability. It is worth noting that curcumin itself is an inhibitor of efflux active transporters, such as BCRP and MRP2, and thus an absorption promoter [62]. This can be achieved by using a suitable polymer as a carrier, which will act as an inhibitor of crystallization. In addition, curcumin is an inhibitor of transport proteins [63,64]. This may act as an additional factor to stimulate absorption and inhibit efflux in the case of an insufficiently high supersaturation state.

In this study, it is expected that the generated supersaturation may increase in vitro permeability through artificial membranes simulating the gastrointestinal tract (GIT) and blood–brain barrier (BBB). For the permeability assessment, we chose the PAMPA model since permeability enhancement relies on supersaturation formation and enhancing passive diffusion. As seen in Table 2, the results show a large permeability increase. The concentration of compounds in the acceptor part of the PAMPA model is considerably improved. In the case of curcumin, in the GIT assay, it was 44,611-fold, 43,713-fold, and 42,515-fold, whereas in the BBB assay, it was 2500-fold, 2478-fold, and 2452-fold for E15, X15, and S15, respectively. For hesperetin, E15, X15, and S15 improved permeability 562-fold, 547-fold, and 531-fold in the GIT assay, while in the BBB assay, an improvement in permeability of 301-fold, 290-fold, and 278-fold, respectively, was noted.

In the literature, one can find reports that for extrusion to be successful, temperatures above the Tg of the polymer and above the melting point of the active compound need to be employed [18,65,66]. In this study, we have shown that the thermal properties of the polymer play a major role in the success of hot-melt extrusion. Reaching the compound’s melting point is not necessary for achieving amorphous solid dispersion via hot-melt extrusion since the components can mix at the molecular level owing to the proper melting behavior of the polymer, which results in an adequate consistency of the extruded blend during the process and enables the dissolution of the active compound into the polymer matrix.

## 4. Conclusions

In this study, we have shown that sweeteners (erythritol, xylitol, and sorbitol) exhibit a plasticizing effect on PVP K30, enabling its use in hot-melt extrusion technology well below its original Tg value. The inconvenience of using these plasticizers may be due to the need for an additional process to ensure good mixing of the polymer blend components at the molecular level. In this study, we used freeze-drying; however, it seems that spray-drying could be successfully used on a larger scale, or a pre-extrusion process aiming to melt and molecularly disperse sweeteners within the PVP K30 matrix. Using a physical mixture may not be sufficient for sweetener–polymer interactions to form and for the sweetener to locate between the polymer chains to exert the plasticizing effect. Through this study, we present a solution to use PVP K30 in hot-melt extrusion at temperatures below its Tg value.

Identifying the relationship between the sweetener content and reduction in Tg values allowed the design of an extrusion polymer blend with the desired thermal properties, which in this study was used to obtain fixed-dose amorphous solid dispersions of curcumin and hesperetin. This approach opened the gates for potential medicinal product development due to the benefits of combining curcumin and hesperetin. The obtained systems were characterized by improved solubility and dissolution rate and thus effective improvement in in vitro biopharmaceutical parameters, which are limited by poor solubility in the case of the tested natural compounds. The improved solubility provided an increase in passive permeability as compared to crystalline compounds, thus confirming excellent bioavailability-enhancing capability. Being aware that the therapeutical success of the curcumin and hesperetin combination is limited due to poor solubility, we hope that the results of this study will serve as a springboard in the future for the development of an effective pharmaceutical, or more precisely, a nutraceutical, based on these plant-origin compounds.

## Figures and Tables

**Table 1:** Results of apparent solubility studies of amorphous solid dispersions and solubility improvement with regard to curcumin and hesperetin.

			Compound			
			Curcumin		Hesperetin	
			Concentration (mg/mL)	Improvement (-Fold)	Concentration (mg/mL)	Improvement (-Fold)
**System**	pH 1.2 HCl	Raw	0.0001 ± 0.00001 ^d^	N/A	0.004 ± 0.001 ^c^	N/A
		E30	0.939 ± 0.008 ^c^	9390	0.924 ± 0.005 ^b^	231
		X30	0.931 ± 0.012 ^c^	9310	0.915 ± 0.008 ^b^	229
		S30	0.922 ± 0.015 ^c^	9220	0.911 ± 0.014 ^b^	228
		E15	1.595 ± 0.013 ^a^	15,950	1.571 ± 0.011 ^a^	393
		X15	1.589 ± 0.007 ^a, b^	15,890	1.567 ± 0.005 ^a^	392
		S15	1.571 ± 0.011 ^b^	15,710	1.563 ± 0.009 ^a^	391
	pH 6.8 phosphate buffer	Raw	0.00014 ± 0.00002 ^c^	N/A	0.005 ± 0.001 ^c^	N/A
		E30	3.203 ± 0.015 ^b^	22,879	3.170 ± 0.015 ^b^	634
		X30	3.192 ± 0.005 ^b^	22,800	3.161 ± 0.008 ^b^	632
		S30	3.185 ± 0.017 ^b^	22,750	3.153 ± 0.021 ^b^	631
		E15	5.934 ± 0.042 ^a^	42,386	5.921 ± 0.036 ^a^	1184
		X15	5.928 ± 0.052 ^a^	42,343	5.912 ± 0.041 ^a^	1182
		S15	5.920 ± 0.025 ^a^	42,286	5.905 ± 0.023 ^a^	1181

The statistically significant values are presented as ^a–d^, with “^a^” being the highest value (*p* < 0.05). N/A means not applicable.

**Table 2:** Results of the in vitro permeability assay for crystalline curcumin and hesperetin as well as amorphous solid dispersions.

			Compound			
			Curcumin		Hesperetin	
			Concentration (mg/mL)	Improvement (-Fold)	Concentration (mg/mL)	Improvement (-Fold)
Model	GIT	Raw	3.34 × 10^−6^ ± 1.94 × 10^−6 c^	N/A	2.58 × 10^−5^ ± 6.16 × 10^−6 c^	N/A
		E30	3.52 × 10^−2^ ± 7.61 × 10^−3 b^	10,539	5.35 × 10^−3^ ± 7.12 × 10^−4 b^	207
		X30	3.47 × 10^−2^ ± 8.41 × 10^−3 b^	10,389	5.27 × 10^−3^ ± 8.84 × 10^−4 b^	204
		S30	3.41 × 10^−2^ ± 4.15 × 10^−3 b^	10,201	5.22 × 10^−3^ ± 4.11 × 10^−4 b^	202
		E15	1.49 × 10^−1^ ± 1.29 × 10^−2 a^	44,611	1.45 × 10^−2^ ± 1.42 × 10^−3 a^	562
		X15	1.46 × 10^−1^ ± 8.57 × 10^−3 a^	43,713	1.41 × 10^−2^ ± 8.15 × 10^−3 a^	547
		S15	1.42 × 10^−1^ ± 2.82 × 10^−2 a^	42,515	1.37 × 10^−2^ ± 2.87 × 10^−3 a^	531
	BBB	Raw	1.86 × 10^−5^ ± 2.48 × 10^−6 c^	N/A	3.89 × 10^−5^ ± 1.66 × 10^−5 c^	N/A
		E30	1.44 × 10^−2^ ± 9.82 × 10^−4 b^	774	2.39 × 10^−3^ ± 9.57 × 10^−5 b^	61
		X30	1.39 × 10^−2^ ± 2.87 × 10^−3 b^	747	2.31 × 10^−3^ ± 2.78 × 10^−4 b^	59
		S30	1.32 × 10^−2^ ± 4.28 × 10^−4 b^	710	2.27 × 10^−3^ ± 4.91 × 10^−5 b^	58
		E15	4.65 × 10^−2^ ± 3.69 × 10^−3 a^	2500	1.17 × 10^−2^ ± 3.79 × 10^−3 a^	301
		X15	4.61 × 10^−2^ ± 7.81 × 10^−4 a^	2478	1.13 × 10^−2^ ± 7.93 × 10^−4 a^	290
		S15	4.56 × 10^−2^ ± 7.15 × 10^−4 a^	2452	1.08 × 10^−2^ ± 7.11 × 10^−4 a^	278

The statistically significant values are presented as ^a–c^, with “^a^” being the highest value (*p* < 0.05). N/A means not applicable.

## Data Availability

Data are contained within the article.

## Supplementary Materials

The following supporting information can be downloaded at: https://www.mdpi.com/article/10.3390/pharmaceutics16050659/s1, Table S1: HPLC method validation parameters. Figure S1: Chromatograms of standards and systems, curcumin standard (a), hesperetin standard (b), system 420 nm (c), and system 288 nm (d).
