# Supplementary material for: Sweeteners Show a Plasticizing Effect on PVP K30—A Solution for the Hot-Melt Extrusion of Fixed-Dose Amorphous Curcumin-Hesperetin Solid Dispersions
Source: Pharmaceutics. 2024 May 15;16(5):659. doi: 10.3390/pharmaceutics16050659 (PMC11124940; doi:10.3390/pharmaceutics16050659)
Supplement: Supplementary file 1 [file pharmaceutics-16-00659-s001.zip › pharmaceutics-3001493-supplementary.pdf]

pharmaceutics

Article

Sweeteners Show a Plasticizing Effect on PVP K30—A Solution for the Hot-Melt Extrusion of Fixed-Dose Amorphous Curcumin-Hesperetin Solid Dispersions

Kamil Wdowiak [1], Lidia Tajber [2], Andrzej Miklaszewski [3] and Judyta Cielecka-Piontek [1,*]

[1] Department of Pharmacognosy and Biomaterials, Poznan University of Medical Sciences, 3 Rokietnicka St., 60-806 Poznan, Poland; kamil.wdowiak@student.ump.edu.pl
[2] School of Pharmacy and Pharmaceutical Sciences, Trinity College Dublin, University of Dublin, D02 PN40 Dublin, Ireland; ltajber@tcd.ie
[3] Institute of Materials Science and Engineering, Poznan University of Technology, Jana Pawla II 24, 61-138 Poznan, Poland; andrzej.miklaszewski@put.poznan.pl
* Correspondence: jpiontek@ump.edu.pl

Citation: Wdowiak, K.; Tajber, L.; Miklaszewski, A.; Cielecka-Piontek, J. Sweeteners Show a Plasticizing Effect on PVP K30—A Solution for the Hot-Melt Extrusion of Fixed-Dose Amorphous Curcumin-Hesperetin Solid Dispersions. *Pharmaceutics* **2024**, *16*, 659. https://doi.org/10.3390/pharmaceutics16050659

Academic Editor: Ecevit Bilgili

Received: 22 April 2024
Revised: 8 May 2024
Accepted: 13 May 2024
Published: 15 May 2024

Copyright: © 2024 by the author. Licensee MDPI, Basel, Switzerland. This article is an open access article distributed under the terms and conditions of the Creative Commons Attribution (CC BY) license (https://creativecommons.org/licenses/by/4.0/).

Chromatographic conditions:

- Stationary phase - Dr. Maisch ReproSil-Pur Basic-C18 100 Å column, 5 µm particle size, 250 × 4.60 mm
- Mobile phase - methanol/0.1% acetic acid (80:20 v/v)
- Column temperature - 30 °C
- Flow rate - 1.0 mL/min

Table S1. HPLC method validation parameters.

**Curcumin**	
Parameter	Curcumin dissolved in methanol<br>Injection volume 10 µl
Linearity range (mg/mL)	0.00004 – 0.2
Correlation coefficient (r)	0.9995
$a \pm S_a$	85245719 ± 2049391
$b \pm S_b$	insignificant (α=0.05)
LOD (mg/mL)	0.0083
LOQ (mg/mL)	0.025
Retention Time	6.644
**Hesperetin**	
Parameter	Hesperetin dissolved in methanol<br>Injection volume 10 µl
Linearity range (mg/mL)	0.00003 – 0.3
Correlation coefficient (r)	0.9999
$a \pm S_a$	80201208 ± 341776
$b \pm S_b$	insignificant (α=0.05)
LOD (mg/mL)	0.0022
LOQ (mg/mL)	0.0068
Retention Time (min)	4.221

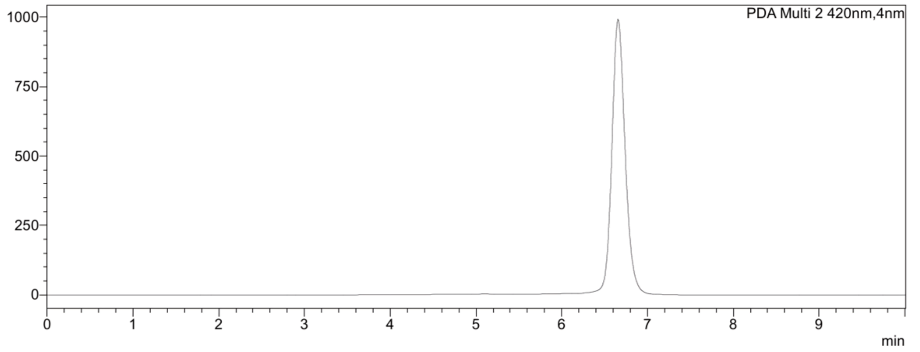

(a)

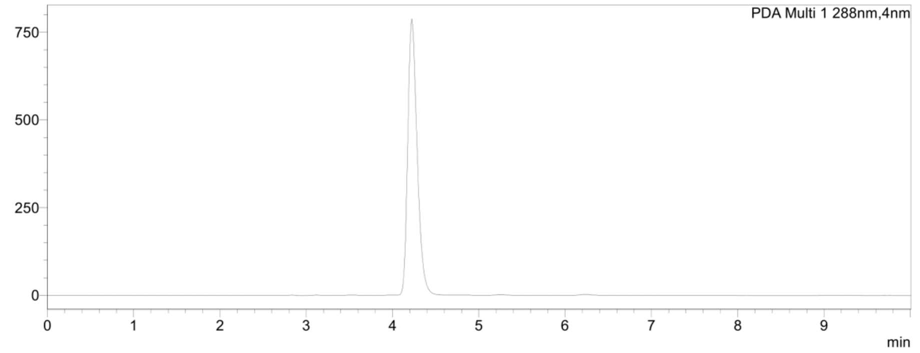

(b)

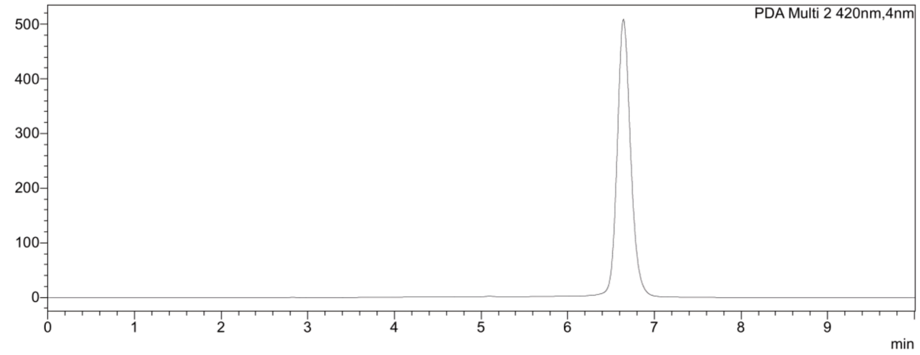

(c)

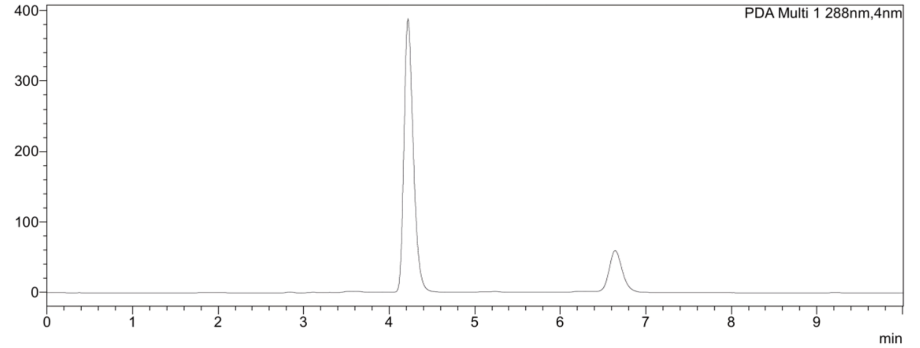

(d)

Figure S1. Chromatograms of standards and systems, curcumin standard (a), hesperetin standard (b), system 420 nm (c), system 288 nm (d).

Disclaimer/Publisher's Note: The statements, opinions and data contained in all publications are solely those of the individual author(s) and contributor(s) and not of MDPI and/or the editor(s). MDPI and/or the editor(s) disclaim responsibility for any injury to people or property resulting from any ideas, methods, instructions or products referred to in the content.
